# Chemokine mediated signalling within arteries promotes vascular smooth muscle cell recruitment

**DOI:** 10.1038/s42003-020-01462-7

**Published:** 2020-12-04

**Authors:** Amber N. Stratman, Margaret C. Burns, Olivia M. Farrelly, Andrew E. Davis, Wenling Li, Van N. Pham, Daniel Castranova, Joseph J. Yano, Lauren M. Goddard, Oliver Nguyen, Marina Venero Galanternik, Timothy J. Bolan, Mark L. Kahn, Yoh-suke Mukouyama, Brant M. Weinstein

**Affiliations:** 1 Division of Developmental Biology, National Institute of Child Health and Human Development, National Institutes of Health, Bethesda, MD 20892 USA; 2 Department of Cell Biology and Physiology, Washington University in St. Louis School of Medicine, St. Louis, MO 63110 USA; 3 Cell and Developmental Biology Center, National Heart, Lung, and Blood Institute, National Institutes of Health, Bethesda, MD 20892 USA; 4 Department of Medicine and Cardiovascular Institute, University of Pennsylvania, 3400 Civic Center Boulevard, Philadelphia, PA 19104 USA

**Keywords:** Angiogenesis, Morphogenesis, Cell signalling

## Abstract

The preferential accumulation of vascular smooth muscle cells (vSMCs) on arteries versus veins during early development is a well-described phenomenon, but the molecular pathways underlying this polarization are not well understood. In zebrafish, the *cxcr4a* receptor (mammalian CXCR4) and its ligand *cxcl12b* (mammalian CXCL12) are both preferentially expressed on arteries at time points consistent with the arrival and differentiation of the first vSMCs during vascular development. We show that autocrine *cxcl12b/cxcr4* activity leads to increased production of the vSMC chemoattractant ligand *pdgfb* by endothelial cells in vitro and increased expression of *pdgfb* by arteries of zebrafish and mice in vivo. Additionally, we demonstrate that expression of the blood flow-regulated transcription factor *klf2a* in primitive veins negatively regulates *cxcr4/cxcl12* and *pdgfb* expression, restricting vSMC recruitment to the arterial vasculature. Together, this signalling axis leads to the differential acquisition of vSMCs at sites where *klf2a* expression is low and both *cxcr4a* and *pdgfb* are co-expressed, i.e. arteries during early development.

## Abstract (web summary)

Stratman et al. provide evidence linking the *cxcl12b/cxcr4a* signaling axis in endothelial cells to an increased release of platelet-derived growth factor b, leading to the recruitment of smooth muscle cells to developing arteries. This signalling axis is suppressed in the venous endothelium during early development by the high expression of blood flow-regulated transcription factor *klf2a*.

## Introduction

Endothelial cells (ECs) and mural cells are the main cellular constituents required for assembly of the arterial vascular wall. ECs form a single cell-thick, lumenized tube that is in direct contact with blood cells, immune cells, and hemodynamic forces. The endothelium is then surrounded by mural cells, including vascular smooth muscle cells (vSMCs) and pericytes—perivascular cell populations that promote long-term vessel stabilization and regulate vascular tone. vSMCs are largely associated with large caliber vessels, in particular arteries, while pericytes are associated with smaller vessels such as those in capillary beds. Arterial-associated vSMCs provide tensile strength to the vascular wall by promoting maintenance and assembly of the vascular basement membrane, and long-term help regulate blood pressure and counter blood flow forces coming out of the heart^1–15^. Although vSMCs are critical modulators of arterial vasculature function, very little is known about the molecular cues that direct their preferential association with arteries rather than veins during early development. This preference has largely been thought to be mediated by differences in blood flow patterns, rates, and shear stresses associated with the arterial vasculature versus the venous vasculature^2,16–21^. Although flow-mediated cues are likely critical, the cellular effectors mediating responses to blood flow and shear stress have yet to be fully elucidated.

The Klf2 transcription factor is one of the best-known blood flow-modulated genes, making it an attractive candidate for a role in flow-based regulation of vSMC recruitment. Klf2 is heavily expressed by postnatal ECs and immune cells, and it has been studied extensively for its connection to atherosclerosis^22–32^. In atherosclerotic disease, sites of low Klf2 expression are prone to lesion development, hinting that suppressed Klf2-mediated signaling is linked to an activated state of cells comprising the vascular wall^25,32–38^. Indeed, in vitro models suggest that EC induction of Klf2 could be a negative regulator of adjacent vSMC motility via promoted cellular differentiation^34,37,39^. Studies in mice, zebrafish, and in vitro have implicated Klf2 in angiogenesis, remodeling of the aortic outflow tract, heart formation and valve formation, and vascular stabilization during development^20,24,27–29,32,37,40–47^. However, the role of Klf2 in vSMC biology and function, and how it interfaces with known pathways and factors regulating vSMC recruitment and differentiation, remains largely unexplored.

Platelet-derived growth factor B (PDGFB) is one of the best-described factors regulating vSMC/mural cell biology. PDGFB ligand is produced by ECs and it signals to mural cells via the PDGFRB receptor. A variety of studies have shown that this signaling pathway is critical for mural cell recruitment and proliferation, including the original reports demonstrating that PDGFRB knockout mice have decreased mural cell coverage of vessels and increased vessel dilation^1,2,6,8,16,48–51^. However, while recruitment of vSMCs is almost exclusively restricted to arteries during early development, PDGFB ligand is initially expressed on both primitive veins and arteries, before becoming largely restricted to arteries^51–54^, suggesting that other factors may help to suppress PDGFB expression in veins to direct vSMC recruitment to arteries.

The CXCR4 chemokine receptor and its associated ligand CXCL12 (aka SDF1a) are both expressed heavily on arteries or in tissue directly adjacent to arteries during early development^20,55–59^. Chemokine signaling has been reported to have effects on vascular development, including during formation of blood vessels, formation of lymphatics, and potentially differentiation and recruitment of mural cells^55,57–67^. Knockout of CXCR4 in mice results in defective arterial patterning in the developing skin, in particular lack of alignment with nerves, with associated defects in mural cell coverage of the vasculature (the effects on large vessels were not analyzed in these studies)^57^. Other studies have suggested more direct effects of chemokine signaling in helping mural cells maintain their de-differentiated status, allowing for increased cellular motility, proliferation, and synthetic/matrix producing phenotype^65,66,68–70^. CXCR4 and PDGFB are both thought to be blood flow responsive genes, suggesting potential links to Klf2^20,58,59^.

In this report, we elucidate a molecular pathway promoting preferential association of vSMC with arterial blood vessels. We demonstrate that CXCL12/CXCR4 chemokine signaling in the arterial endothelium promotes PDGFB ligand expression, driving arterial vSMC recruitment. We also show that venous expression of KLF2 after the onset of blood flow negatively regulates CXCR4 and PDGFB production to inhibit venous recruitment of vSMC. Altogether, our findings highlight a molecular pathway driving arterial-specific recruitment of vSMCs, via regulated and coupled expression of chemokines and PDGFB in the vasculature.

## Results

### Smooth muscle cells preferentially associate with developing arteries

**Fig. 1:**
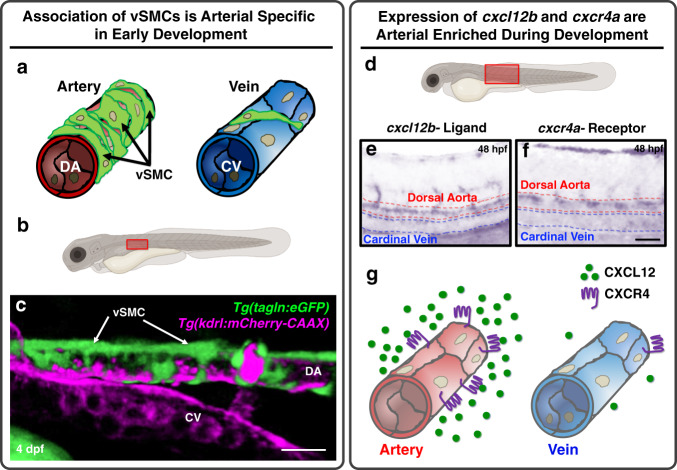
vSMCs associate with arteries during development. **a** Schematic diagram illustrating vSMC coverage of arteries and lack of coverage of veins. **b** Schematic diagram of a zebrafish larva with the red box highlighting the area imaged in panel **c**. **c** Confocal micrograph of the anterior trunk of a 4 dpf *Tg(tagln:eGFP), Tg(kdrl:mCherry-CAAX)* double-transgenic zebrafish larva expressing eGFP in vSMCs (green) and mCherry-CAAX in the endothelium (magenta)^2^. vSMCs are associated with the dorsal aorta (DA) and not the cardinal vein (CV). **d** Schematic diagram of a zebrafish larva with the red box highlighting the area imaged in panels **e** and **f**. **e**, **f** Whole mount in situ hybridization of the mid-trunk of 48 hpf zebrafish larvae probed for *cxcl2b* ligand (**e**) or *cxcr4a* receptor (**f**). Expression of both genes is enriched in the dorsal aorta compared to the cardinal vein. *n* = 20; scale bars = 75 µm. **g** Schematic representation of arterial-enriched expression of *cxcl12b* and *cxcr4a*.

The presence of a thickened vascular wall with abundant vascular smooth muscle cells (vSMC) is perhaps the most clearly evident morphological feature distinguishing arteries from veins, but the molecular mechanisms responsible for preferential acquisition of vSMCs by arteries remains largely unexplored. As we and others have previously reported, vSMCs emerge from the medial sclerotome of the zebrafish trunk beginning at ~3 days post fertilization (dpf), taking up residence around the closely juxtaposed dorsal aorta, but not around the equally close cardinal vein (Fig. 1a)^2,10,18,71^. This differential recruitment of vSMCs to the trunk dorsal aorta but not the cardinal vein is readily observed in 4–6 dpf *Tg(tagln:eGFP),Tg(kdrl:mCherry-CAAX)* double-transgenic zebrafish with mCherry-positive vascular endothelium (magenta) and eGFP-positive vSMCs (green) (Fig. 1b, c)^2^. We identify these cells as vSMCs based on their localization to the dorsal aorta and on our previous work showing that they form a multilayered wall of vSMCs over time^2^.

To identify candidate factors that might be playing a role in guiding the dorsal aorta-restricted recruitment of vSMCs, we used the ZFIN gene expression database (zfin.org) to carry out an in silico survey for secreted factors differentially expressed in the dorsal aorta but not in the cardinal vein at ~2 dpf (details described in the “methods”). The *cxcl12b* chemokine ligand (also known as SDF1a) and its receptor *cxcr4a* were identified as promising candidates. Although expression is present in both cardinal vein and dorsal aorta at earlier stages of development (24–34 h post fertilization, hpf; Supplementary Fig. 1), by 2.5–3 dpf when vSMC recruitment is beginning^2,10,18,71^, *cxcl12b* and *cxcr4a* expression is strongly enriched in the dorsal aorta compared to the cardinal vein (Fig. 1d–g).

### Chemokine signaling regulates vSMC association with arteries

**Fig. 2:**
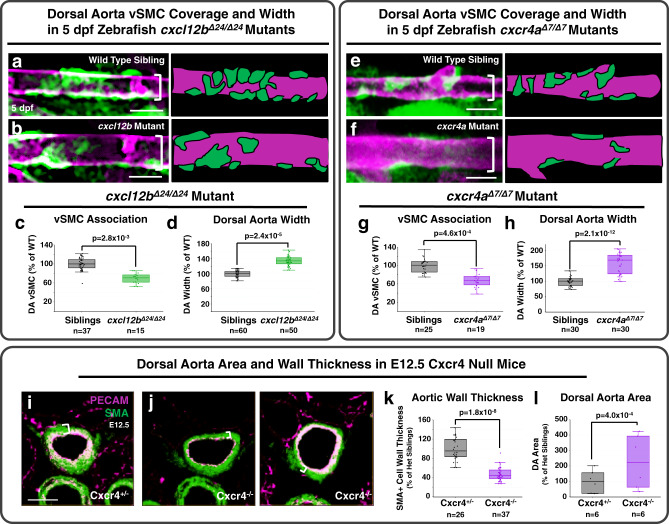
Disrupting *cxcl12b/cxcr4a* signaling decreases vSMC association with arteries. **a**, **b** Confocal images (left) and schematic representations (right) of the dorsal aorta (DA) in the anterior trunk of 5 dpf *Tg(tagln:eGFP), Tg(kdrl:mCherry-CAAX)* double-transgenic sibling (**a**) or *cxcl12b*^*Δ24/Δ24*^ mutant (**b**) zebrafish expressing eGFP in vSMCs (green) and mCherry-CAAX in the endothelium (magenta). **c**, **d** Quantification of the number of associated vSMC (**c**) and width (**d**) of the dorsal aorta in 5 dpf *Tg(tagln:eGFP), Tg(kdrl:mCherry-CAAX)* double-transgenic sibling (black columns; *n* = 37 (**c**) and *n* = 60 (**d**)) or *cxcl12b*^*Δ24/Δ24*^ mutant (green columns; n = 15 (**c**) and *n* = 50 (**d**)) larvae. Values are expressed as a percentage of control siblings and averaged from three individual experiments. **e**, **f** Confocal images (left) and schematic representations (right) of the dorsal aorta (DA) in the anterior trunk of 5 dpf *Tg(tagln:eGFP)*, *Tg(kdrl:mCherry-CAAX)* double-transgenic siblings (**e**) or *cxcr4*^*Δ7/Δ7*^ mutant (**f**) zebrafish expressing eGFP in vSMCs (green) and mCherry-CAAX in the endothelium (magenta). The schematic representations demonstrate what were counted as cells in the adjacent panels. **g**, **h** Quantification of the number of associated vSMC (G) and width (**h**) of the dorsal aorta in 5 dpf *Tg(tagln:eGFP), Tg(kdrl:mCherry-CAAX)* double-transgenic sibling (black columns; *n* = 25 (**g**) and *n* = 30 (**h**)) or *cxcr4*^*Δ7/Δ7*^ mutant (purple columns; *n* = 19 (**g**) and *n* = 30 (**h**)) larvae. Values are expressed as a percentage of control siblings and averaged from three individual experiments. **i**, **j** Confocal images of immunohistochemically stained transverse sections through the dorsal aorta of E12.5 Cxcr4^+/−^ heterozygous sibling (**i**) and Cxcr4^−/−^ mutant (**j**) mice, probed for platelet endothelial cell adhesion molecule-1 (PECAM) for endothelium (blue) and alpha smooth muscle actin (SMA) for vascular smooth muscle (vSMC, red). White brackets note the thickness of the vSMC layer surrounding the DA. **k**, **l** Quantification of aortic wall thickness (**k**) and lumenal area (**l**) of E12.5 Cxcr4^+/−^ heterozygous sibling (black columns; *n* = 26 (**k**) and *n* = 6 (**l**)) and Cxcr4^−/−^ mutant (purple columns; *n* = 37 (**k**) and *n* = 6 (**l**)) mice, measured from immunohistochemically stained sections as in panels **i** and **j**. The range for wall thickness was defined by the inner and outermost SMA staining detected in the images. Values are expressed as a percentage of heterozygous siblings and averaged from three individual experiments. Scale bars = 75 µm (panels **a**, **b**, **e**, **f**), 400 µm (panels **i**, **j**). Box plots are graphed showing the median versus the first and third quartiles of the data (the middle, top, and bottom lines of the box, respectively). The whiskers demonstrate the spread of data within 1.5x above and below the interquartile range. All data points are shown as individual dots, with outliers shown above or below the whiskers. *P*-values are indicated above statistically significant datasets and were generated using student’s *t*-tests.

**Fig. 3:**
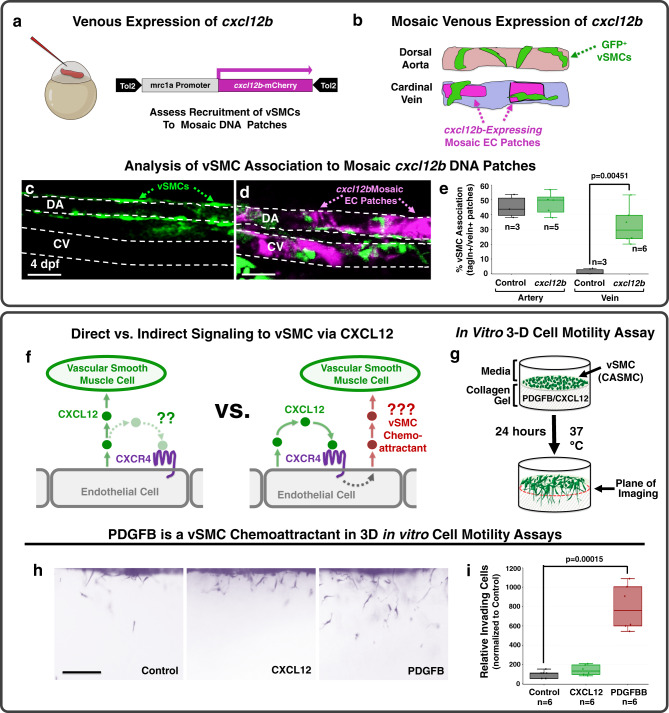
*cxcl12b* promotes vSMC association without serving as a direct chemoattractant. **a**, **b** Schematic diagrams illustrating the experimental design for using the *mrc1a* promoter to drive ectopic mosaic expression of *cxcl12b* in veins. **a** A *Tol2(mrc1a:cxcl12b-2a-mCherry)* DNA construct co-translationally expressing *cxcl12b* and mCherry under the control of the *mrc1a* promoter is injected into *Tg(tagln:eGFP)* transgenic zebrafish embryos at the 1 cell stage. **b** At 4 dpf *tol2(mrc1a:cxcl12b-2a-mCherry)*-injected zebrafish larvae are analyzed for vSMC (eGFP) association at sites of mCherry (i.e., *cxcl12b*) expression in the dorsal aorta and cardinal vein. **c**, **d** Representative confocal images of the mid-trunk of 4 dpf *Tg(tagln:eGFP)* transgenic larvae injected with either control *Tol2(mrc1a)* “empty vector” (**c**) or *Tol2(mrc1a:cxcl12b-2a-mCherry)* (**d**). eGFP-expressing vSMCs are shown in green, *cxcl12b-2a-mCherry* expression in dorsal aorta (DA) or cardinal vein (CV) endothelium is shown in magenta. **e** Quantification of eGFP-positive vSMC associated with the dorsal aorta (DA) or cardinal vein (CV) in 4 dpf *Tg(tagln:eGFP)* transgenic zebrafish injected with either control *Tol2(mrc1a)* “empty vector” (black columns; *n* = 3 for artery and vein) or *Tol2(mrc1a:cxcl12b-2a-mCherry)* (green columns; *n* = 5 artery, *n* = 6 vein), showing strongly increased association of vSMCs with the cardinal vein. **f** Schematic diagrams showing potential models for direct (left) versus indirect (right) mechanisms for promoting arterial recruitment of vSMC via CXCL12. **g** Schematic diagram illustrating the 3-D coronary artery smooth muscle cell (CASMC) motility assay. CXCL12, PDGFB, or nothing (control) is placed within the collagen gel to determine if CASMCs migrate towards these potential chemoattractants. **h** Representative lateral images of 3-D collagen gels showing CASMCs within the collagen matrix for each gel condition. **i** Quantification of the relative number of CASMCs invading the collagen gel. The control is set to 100% and the CXCL12 and PDGFB conditions normalized to this level of invasion. Scale bars = 75 µm (panels **c**, **d**), 200 µm (panel **h**); *n* = 6. Box plots are graphed showing the median versus the first and third quartiles of the data (the middle, top, and bottom lines of the box, respectively). The whiskers demonstrate the spread of data within 1.5x above and below the interquartile range. All data points are shown as individual dots, with outliers shown above or below the whiskers. *P*-values are indicated above statistically significant datasets. Statistics in panels **e** and **i** were run using two-way ANOVA to calculate *P*-values.

To examine whether *cxcl12b/cxcr4a* chemokine signaling plays a role in dorsal aorta-specific vSMC recruitment, we used CRISPR/Cas9 technology to generate 24 bp and 7 bp deletion mutants in *cxcl12b* and *cxcr4a*, respectively (Supplementary Fig. 2A, B). Homozygous *cxcl12b*^*Δ24/Δ24*^ mutant animals show reduced numbers of vSMCs associated with the dorsal aorta at 5 dpf (Fig. 2a–c and Supplementary Table 1) and an increased dorsal aorta diameter (Fig. 2d), a previously reported consequence of defects in vSMC coverage of vessels^2,3^. Homozygous *cxcr4a*^*Δ7/Δ7*^ mutant animals also have reduced numbers of dorsal aorta-associated vSMCs and increased dorsal aorta diameter (Fig. 2e–h and Supplementary Table 1). Deletion of the Cxcr4 gene in mice similarly results in decreased thickness of the vSMC—smooth muscle actin-positive—wall of the dorsal aorta (Fig. 2i–k) and dorsal aorta luminal enlargement (Fig. 2l) at E12.5, suggesting the role of chemokine signaling in vSMC recruitment is evolutionarily conserved. If chemokine signaling is indeed promoting vSMC recruitment to the dorsal aorta, we reasoned that forced mis-expression of *cxcl12b* in the cardinal vein (where it is not normally expressed at 3 dpf, Supplementary Fig. 1) might result in ectopic targeting of vSMCs to this vessel (Fig. 3a, b). To test this, we injected single cell *Tg(tagln:eGFP)* transgenic zebrafish with a Tol2 transgene containing the *mrc1a* promoter driving expression of *cxcl12b* and mCherry, co-translationally linked together via the 2A peptide sequence (Fig. 3a, b). We have previously shown that the *mrc1a* promoter drives robust expression in the early cardinal vein across all stages of vascular development, in addition to modest expression in other vessels until about 4 dpf^72^. Analysis at 4 dpf of mosaic *cxcl12b* patches in the cardinal vein (marked by mCherry expression) shows ectopic cardinal vein recruitment of vSMCs without affecting vSMC acquisition by the dorsal aorta (Fig. 3c–e). By this time point in development a substantial number of vSMCs have been recruited to the dorsal aorta but there are typically still no vSMCs associated with the cardinal vein in control animals. Together these gain- and loss-of-function experiments support the idea that dorsal aorta-restricted chemokine signaling is important for differential arterial recruitment of vSMCs.

### PDGFB modulates vSMC association with the dorsal aorta

**Fig. 4:**
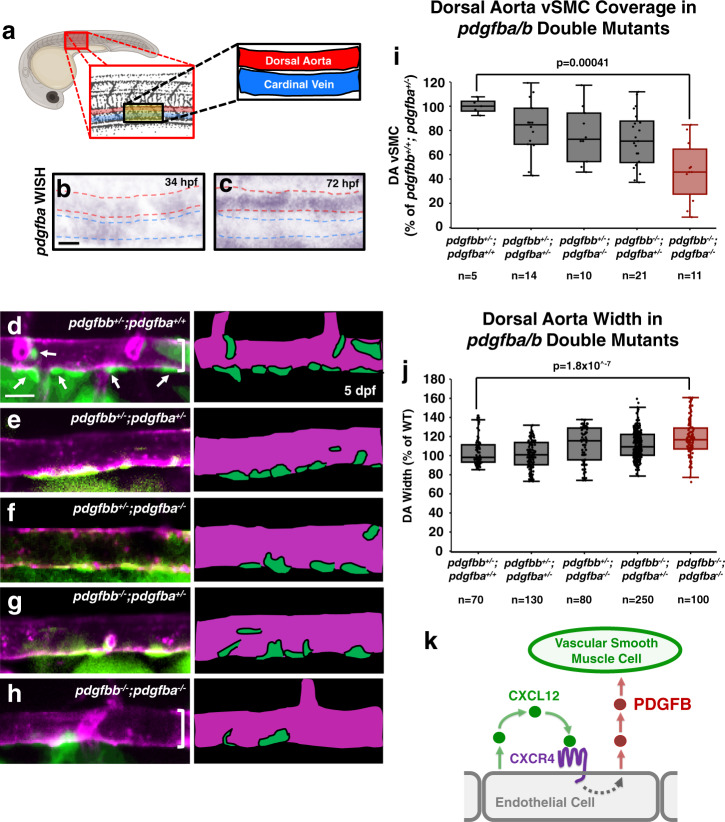
*pdgfb*-mediated signaling regulates vSMC association with arteries. **a** Schematic representation of the area imaged for in situ hybridization analysis, demonstrating the location of the dorsal aorta (DA) and cardinal vein (CV) within these images. **b**, **c** Whole mount in situ hybridization (WISH) images of *pdgfba* transcript (purple) in 34 hpf (**b**) and 72 hpf (**c**) zebrafish. Red dashed lines outline the dorsal aorta, blue dashed lines outline the cardinal vein. An expanded time course including these images can be found in Supplemental Fig. 1. **d**–**h** Confocal images (left) and schematic representations (right) of the dorsal aorta (DA) in the anterior trunk of 5 dpf *Tg(tagln:eGFP), Tg(kdrl:mCherry-CAAX)* double-transgenic *pdgfbb*^+/−^*, pdgfba*^+/+^ sibling (**d**); *pdgfbb*^+/−^*, pdgfba*^+/−^ (**e**); *pdgfbb*^+/−^*, pdgfba*^−/−^ (**f**); *pdgfbb*^−/−^*, pdgfba*^+/−^ (**g**); or, *pdgfbb*^−/−^, *pdgfba*^−/−^ double homozygous mutant (**h**) zebrafish expressing eGFP in vSMCs (green) and mCherry-CAAX in the endothelium (magenta). The schematic representations demonstrate what were counted as cells in the adjacent panels. Scale bars = 75 µm. **i**, **j** Quantification of the number of associated vSMCs (**i**) and width (**j**) of the dorsal aorta in 5 dpf *Tg(tagln:eGFP), Tg(kdrl:mCherry-CAAX)* double-transgenic zebrafish carrying different combinations of heterozygous or homozygous *pdgfba* and *pdgfbb* mutants. Values are averaged from three individual experiments and expressed as a percentage of the *pdgfbb*^+/−^, *pdgfba*^+/+^ control. Total *n* number per condition is shown below each sample in the graphs. **k** Schematic diagram illustrating the proposed model for endothelial-autonomous chemokine signaling driving increased endothelial PDGFB ligand production, and thereby indirectly promoting vSMC acquisition by arteries. Box plots are graphed showing the median versus the first and third quartiles of the data (the middle, top, and bottom lines of the box, respectively). The whiskers demonstrate the spread of data within 1.5x above and below the interquartile range. All data points are shown as individual dots, with outliers shown above or below the whiskers. *P*-values are indicated above statistically significant datasets and were calculated using one-way ANOVA.

We carried out additional experiments to examine whether CXCL12 acts directly as a paracrine chemoattractant for arterial vSMCs or indirectly via autocrine activation of arterial endothelial CXCR4 to induce expression of some other chemoattractant factor (Fig. 3f). Using a previously described in vitro three-dimensional (3-D) cell motility assay (Fig. 3g)^3,63,73^, we showed that human coronary artery smooth muscle cells (CASMCs) have little or no chemoattractant activity for CXCL12, unlike PDGFB, which has a robust activity in this assay (Fig. 3h, i). PDGFB is a well-documented in vivo vSMC chemoattractant expressed by ECs and required for vSMC recruitment in mice^6,50,51^. Zebrafish *pdgfba* is expressed weakly in both the dorsal aorta and cardinal vein up to approximately 48 hpf (Fig. 4a–c and Supplementary Fig. 1), but by the time vSMC recruitment begins at 72 hpf it shows preferential expression in the developing dorsal aorta (Fig. 4c). To examine the role of *pdgfb* in vSMC recruitment in the zebrafish we used CRISPR/Cas9 mutagenesis to generate mutants in the *pdgfbb* and closely related *pdgfba* genes (Supplementary Fig. 2C). Zebrafish homozygous mutants for either *pdgfbb* or *pdgfba* alone display modest reductions in vSMC recruitment to the dorsal aorta at 5 dpf, but animals homozygous for the mutations in both genes display a strong reduction in vSMC recruitment and widening of the dorsal aorta (Fig. 4d–j and Supplementary Table 1). The single *pdgfba*^−/−^ and *pdgfbb*^−/−^ mutants do not have significant phenotypes and the *pdgfbb*^−/−^ mutants are homozygous viable. We thus crossed *pdgfbb*^−/−^; *pdgfba*^+/−^ zebrafish to *pdgfbb*^+/−^; *pdgfba*^+/−^ zebrafish to increase recovery of double homozygous mutants for analysis. Therefore, there are no pure wild type siblings in this data set. Altogether with previous findings from our lab using expression of dominant-negative *pdgfrb*-DN to disrupt vSMC recruitment^2^, these results suggest that, as noted in mice, PDGFB signaling serves as an important vSMC chemoattractant in the zebrafish.

### Chemokine signaling regulates expression of PDGFB

**Fig. 5:**
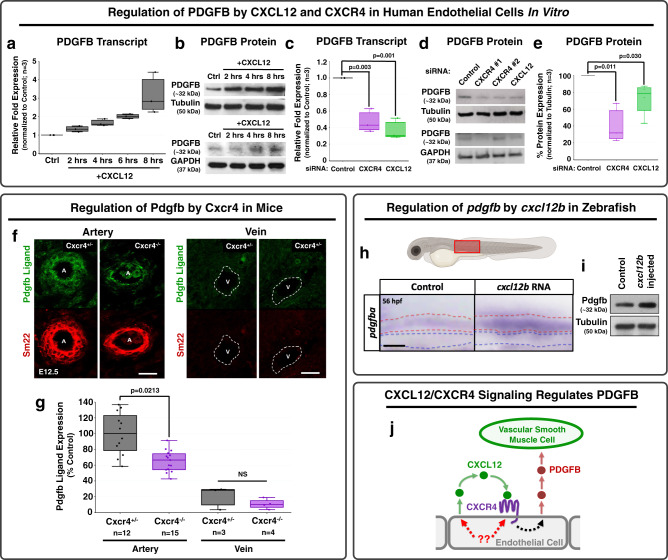
Chemokine signaling regulates PDGFB transcript and protein levels across species. **a**, **b** PDGFB transcript (**a**) and protein (**b**) in HUVEC cells cultured in vitro in a confluent cell monolayer for up to 8 h with (“+CXCL12”) or without (“CTRL”) added recombinant CXCL12. Relative PDGFB transcript levels (**a**) and protein levels (**b**) were measured by qPCR and Western blot, respectively, showing an upregulation of both PDGFB transcript and PDGFB protein levels in response to stimulation by CXCL12. The PDGFB western blots are shown in comparison to two loading controls, tubulin and GAPDH. **c**–**e** PDGFB transcript (**c**) and protein levels (**d**, **e**) in HUVEC cells cultured in vitro in a confluent cell monolayer and treated with either control, CXCR4 (target #1 or target #2- all experimental quantification utilizes siRNA target #1), or CXCL12 siRNAs. Relative PDGFB transcript (**c**) and protein (**e**, **f**) levels were measured by qPCR and western blot, respectively, showing suppression of both PDGFB transcript and protein in response to either CXCR4 or CXCL12 knockdown. The PDGFB western blots are shown in comparison to two loading controls, tubulin (top) and GAPDH (bottom), and the quantification is normalized to tubulin expression. Values in **a**, **c**, and **e** are representative of three individual experiments and expressed as a percentage of control. Samples are generated by pooling 20 individual embryos per condition. (**a**, **c**). *n* = 3 for all in vitro experiments. **f** Confocal images of immunohistochemically stained transverse sections through large arteries and large veins of E12.5 Cxcr4^+/−^ heterozygous sibling (left) and Cxcr4^−/−^ mutant (right) mice, probed for platelet-derived growth factor B (PDGFB; green) and for smooth muscle 22 alpha (SM22, aka transgelin) for vascular smooth muscle cells (vSMC, red). **g** Quantification of relative PDGFB protein expression in Cxcr4^+/−^ heterozygous embryos (black bars; *n* = 12 arteries, *n* = 3 veins) versus Cxcr4^−/−^ homozygous mutant embryos (purple bars; *n* = 15 arteries, *n* = 4 veins). Values are expressed as a percentage of the heterozygous control condition and images were acquired from three to five individual mice per condition. **h** Schematic diagram of a zebrafish larva with the red box highlighting the area imaged in the lower panels. Lower panels: Whole mount in situ hybridization of the mid-trunk of 56 hpf zebrafish injected with control (left) or *cxcl12b* (right) RNA at the one-cell stage, showing upregulation of *pdgfba* transcript in response to exogenous *cxcl12b*. Red and blue dotted lines in the panels indicate the dorsal aorta and cardinal vein, respectively. Images are representative of 20 embryos. **i** Western blot of whole embryo protein lysate from 54 hpf zebrafish injected with either control (left) or *cxcl12b* (right) RNA, probed for *pdgfb* (top) or alpha tubulin (bottom), showing upregulation of *pdgfb* protein levels in response to exogenous overexpression of *cxcl12b*. Images are representative of data from three individual experiments. **j** Schematic diagram illustrating the proposed model for endothelial-autonomous chemokine signaling driving increased endothelial PDGFB ligand production, thereby indirectly promoting vSMC acquisition by arteries. Putative upstream regulators of CXCL12 and CXCR4 are noted in red. Scale bars = 100 µm (**f**) and 50 µm (**h**). Box plots are graphed showing the median versus the first and third quartiles of the data (the middle, top, and bottom lines of the box, respectively). The whiskers demonstrate the spread of data within 1.5x above and below the interquartile range. All data points are shown as individual dots, with outliers shown above or below the whiskers. *P*-values are indicated above statistically significant datasets. Statistics in panels **c**, **e** were run using one-way ANOVA and **g** using two-way ANOVA to calculate the *P*-values.

To test the possibility that CXCL12/CXCR4 signaling acts indirectly on vSMC motility via autocrine upregulation of PDGFB in arterial endothelium (Fig. 4k), we examined whether chemokine signaling regulates PDGFB transcript and/or protein levels in HUVECs in vitro (Fig. 5a–e). Application of exogenous recombinant CXCL12 ligand to HUVECs in culture results in increased levels of PDGFB transcript (Fig. 5a) and PDGFB protein (Fig. 5b, compared to two different loading controls). Conversely, small-interfering RNA (siRNA) knockdown of endogenous CXCR4 or CXCL12 in cultured HUVECs results in decreased PDGFB transcript levels (Fig. 5c and Supplementary Fig. 3) and decreased PDGFB protein levels (Fig. 5d, e and Supplementary Fig. 3, compared to two different loading controls). To determine whether Cxcr4 is also required for Pdgfb expression in the endothelium in vivo, we used immunostaining to examine Pdgfb expression in the developing arteries of Cxcr4 knockout mice. Compared to their heterozygous siblings, Cxcr4^−/−^ homozygous null mice showed a strong reduction in arterial expression of Pdgfb ligand, as well as reduced vSMC vessel wall thickness as assessed by Sm22^+^ staining at E12.5 (Fig. 5f, g). Expression of Pdgfb in veins was not extensively noted in either heterozygous siblings or Cxcr4^−/−^ homozygous null mice (Fig. 5f, g). Conversely, overexpression of *cxcl12b* ligand in zebrafish larvae via injection of *cxcl12b* RNA resulted in an increase in *pdgfba* transcript (Fig. 5h) and protein levels (Fig. 5i) as assessed by in situ hybridization and western blotting, respectively. Altogether, these results suggest that autocrine CXCL12/CXCR4 chemokine signaling up-regulates PDGFB ligand in endothelial cells to promote recruitment of vSMCs (Fig. 5j).

### KLF2 regulates vSMC recruitment upstream from chemokine signaling

**Fig. 6:**
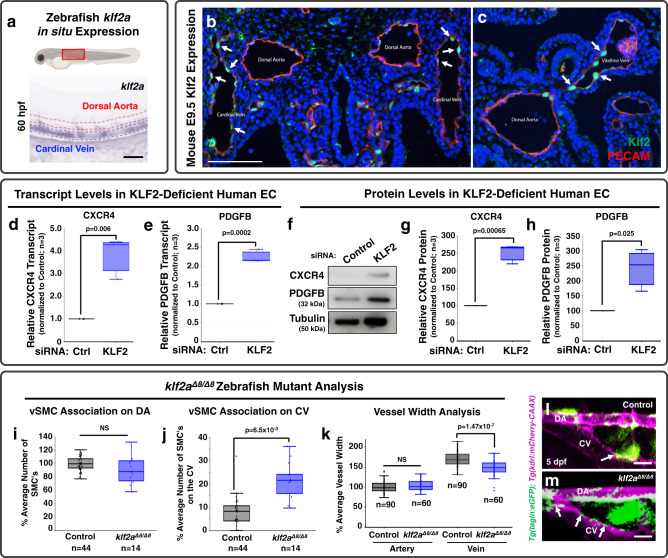
KLF2 is a negative regulator of chemokine signaling and PDGFB during early development. **a** Top: Schematic diagram of a zebrafish larva with the red box highlighting the area imaged below. Bottom: Representative whole mount in situ hybridization (WISH) image of a 60 hpf zebrafish probed for *klf2a*. Red dashed lines indicate the dorsal aorta; blue dashed lines indicate the cardinal vein. **b**, **c** Fluorescent images of transverse sections through E9.5 mice: IHC for GFP was done to amplify signal from a Klf2-GFP knockin allele where the GFP is fused to the N-terminus of Klf2 (Klf2; green) and for platelet endothelial cell adhesion molecule-1 (PECAM) to mark the endothelium (red). Nuclei are labeled with DAPI (blue). Arrows highlight Klf2-positive endothelial nuclei in the cardinal vein (**b**) and vitelline vein (**c**)^32,84^. **d**, **e** Quantitative qPCR measurement of CXCR4 (**d**) and PDGFB (**e**) transcript levels in HUVEC cells cultured in vitro as a confluent cell monolayer and treated with either control (black columns) or KLF2 (blue columns) siRNA. Values are expressed as a percentage of control and are representative of three individual experiments. Samples are generated by pooling 20 individual embryos per condition. **f**–**h** Representative western blot images of CXCR4 and PDGFB protein levels (**f**), and quantification of relative CXCR4 (**g**) and PDGFB protein levels (**h**) from HUVEC cells cultured in vitro in a confluent cell monolayer and treated with either control or KLF2 siRNA. Values in **g**, **h** are averaged from three individual experiments and expressed as a percentage of control. **I, J** Quantification of vSMC number associated with the dorsal aorta (**i**) or cardinal vein (**j**) in the mid-trunk of 5 dpf control wild type/heterozygous siblings (black columns; *n* = 44 (**i**, **j**)) or *klf2a*^*Δ8/Δ8*^ mutant (blue columns; *n* = 14 (**i**, **j**)) animals. Values are averaged from data collected from three separate experiments and are expressed as a percentage of the control sibling average. **k** Quantification of dorsal aorta (DA, left columns) and cardinal vein (CV, right columns) width in the mid-trunk of 5 dpf control sibling (black columns; *n* = 90) or *klf2a*^*Δ8/Δ8*^ mutant (blue columns; *n* = 60) animals. Values are averaged from data collected from three separate experiments and are expressed as a percentage of the average artery width in the control siblings. **l**, **m** Confocal images of the anterior trunk of 5 dpf *Tg(tagln:eGFP), Tg(kdrl:mCherry-CAAX)* control sibling (**l**) and *klf2a*^*Δ8/Δ8*^ mutant (**m**) zebrafish embryos with eGFP-positive vSMCs (green) and mCherry-CAAX-positive endothelial cells (magenta). Arrows point to vSMCs associated with the CV in control versus *klf2a*^*Δ8/Δ8*^ mutants. Scale bars = 300 µm (panels **b**, **c**) and 75 µm (panels **l**, **m**). Box plots are graphed showing the median versus the first and third quartiles of the data (the middle, top, and bottom lines of the box, respectively). The whiskers demonstrate the spread of data within 1.5x above and below the interquartile range. All data points are shown as individual dots, with outliers shown above or below the whiskers. *P*-values are indicated above statistically significant datasets. Statistics in panels **d**, **e** were generated via two-way ANOVA and panel **k** were run using two-way ANOVA; all other panels employ student’s t-tests to calculate the *P*-values.

We next sought to understand what might be responsible for preferentially restricting CXCL12/CXCR4 signaling and thus Pdgfb to the dorsal aorta rather than the cardinal vein. Our primary candidate gene of interest was Klf2—a well-documented blood flow-regulated transcription factor, with reported links to chemokine signaling. In the zebrafish, *klf2a* begins to be expressed in the cardinal vein just after the onset of blood flow between 24 and 32 hpf, and it remains expressed predominantly by veins through at least 72 hpf (Fig. 6a and Supplementary Fig. 1). Preferential expression of Klf2 in primitive veins is also noted in E9.5 mice (Fig. 6b, c), with Klf2 expression enriched in both the cardinal vein (B) and the vitelline vein (C) as compared to the dorsal aorta. These results, and previous reports that KLF2 can negatively regulate chemokine signaling^20^, suggested to us that KLF2 expression in primitive veins might act as a “STOP” signal to block CXCL12/CXCR4 and PDGFB expression in the cardinal vein, thus restricting vSMC recruitment to the dorsal aorta. If this were the case, we predicted removing KLF2 would increase chemokine signaling and PDGFB expression to promote ectopic venous recruitment of vSMCs.

To test this idea, we used siRNA knockdown to suppress KLF2 in HUVECs in vitro and genetic mutants to “knock out” *klf2a* in the zebrafish in vivo. Suppressing KLF2 in HUVECs in vitro led to upregulation of CXCR4 and PDGFB transcript and protein levels (Fig. 6d–h and Supplementary Fig. 3). To examine the consequences of reduced KLF2 in the endothelium in vivo, we used CRISPR/Cas9 mutagenesis to generate an 8 bp deletion mutant in zebrafish *klf2a* (Supplementary Fig. 2D). Although there was no change in the number of vSMCs associated with the dorsal aorta in homozygous *klf2a*^*Δ8/Δ8*^ mutants (Fig. 6i, l, m and Supplementary Table 1), there was a clear increase in the number of vSMCs associated with the cardinal vein (Fig. 6j, l, m). This increase was accompanied by a modest decrease in the diameter of the cardinal vein without any change in the dorsal aorta (Fig. 6k), consistent with the role of vSMCs in regulating vessel diameter. Analysis of *pdgfba* transcript levels on the *klf2a*^*Δ8/Δ8*^ mutant background, reveals maintained expression of *pdgfba* in the cardinal vein of *klf2a*^*Δ8/Δ8*^ mutants that is absent in wild type and heterozygous siblings (Supplementary Fig. 4A). Importantly, these mutants have normal blood flow and heart rates compared to control siblings (Supplementary Fig. 4A and Supplementary Videos 1 and 2). Further, stopping blood flow transiently in the zebrafish supports the concept of this pathway being linked to hemodynamic forces (Supplementary Fig. 5). When blood flow is restricted for 12 h starting at 36 hpf using the chemical BDM (2,3-butanedione monoxime), we see a marked downregulation of *klf2a* transcript, and an associated upregulation of *cxcr4a* and *pdgfba* transcript (Supplementary Fig. 4). Altogether, these results suggest that expression of KLF2 restricts chemokine/PDGFB expression and association of vSMCs to primitive veins during vascular development.

## Discussion

**Fig. 7:**
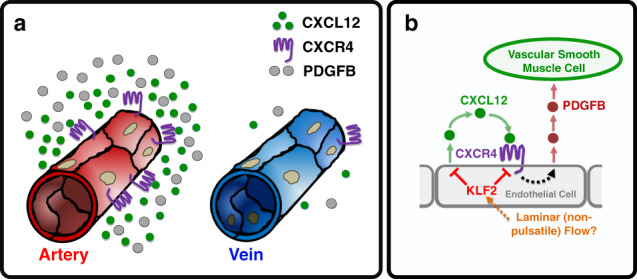
A proposed model for the preferential recruitment of vascular smooth muscle cells to arteries. **a** During the early development of arterial and venous blood vessels the CXCL12 ligand, its receptor CXCR4, and the vascular smooth muscle cell (vSMC) chemoattractant PDGFB are all more highly expressed on arteries than on veins. In contrast, KLF2 is more highly expressed on primitive veins than arteries. **b** A proposed molecular pathway for preferential recruitment of vSMCs to arteries. In arteries, autocrine activation of endothelial CXCR4 by its CXCL12 ligand results in increased production of PDGFB by arterial endothelium, promoting vSMC recruitment to arteries. Expression of KLF2 in primitive veins suppresses expression of CXCL12 and CXCR4, preventing upregulation of PDGFB and limiting vSMC recruitment to veins. The cues directing preferential expression of KLF2 in veins remain unclear, but may involve different types of flow (i.e., pulsatile vs. laminar).

The preferential association of vascular smooth muscle cells with arteries has been appreciated for centuries, but the molecular mechanisms underlying this preference have not been fully elucidated. We have uncovered a molecular pathway that promotes association of vSMCs with developing arteries and reduces vSMC association with veins (Fig. 7). The known blood flow-regulated transcription factor KLF2 helps demarcate and maintain a pro-vSMC state in the endothelium. Sustained expression of KLF2 (zebrafish *klf2a*) in primitive venous endothelium during early development represses expression of *cxcl12b* and *cxcr4a* and autocrine/juxtacrine endothelial chemokine signaling, thereby reducing chemokine signaling-promoted production of *pdgfb* and venous recruitment of vSMC. In contrast, downregulation of *klf2a* in the early arterial endothelium permits expression of *cxcl12b* and *cxcr4a*, restricting production of arterial *pdgfb* and arterial recruitment of vSMCs (Fig. 7). As predicted by this model, either artificially suppressing *klf2a* expression or mis-expressing *cxcl12b* in venous endothelium promotes ectopic venous recruitment of vSMCs (Figs. 3a–e and 6j, l, m).

Altogether, our findings and previous reports describing flow-mediated regulation of KLF2 suggest that changes in blood flow velocity, force, or directionality influence stabilization of the arterial blood vessel wall by modulating the expression of this key transcription factor. In the zebrafish trunk, *klf2a* expression is initially equivalently low in the endothelial cords that will give rise to the dorsal aorta (DA) and cardinal vein (CV), but as these vessels lumenize and circulation begins, the expression of *klf2a* is strengthened in the CV. Since flow begins in the DA and CV at the same time during early development, why do these two vessels show such different *klf2a* expression, and how could flow be playing a role in this difference? Although further work will be needed to definitively answer this question, the *type* of flow that each of these vessels experience is likely playing a critical role in their differential expression of *klf2a*. There is relatively even, constant laminar flow through the CV at these early developmental stages, while the DA experiences a much more pulsatile flow regime. Pulsatile flow results in cyclical changes in velocity and shear, and in the circumferential “stretch” experienced by the endothelial cells in the DA, that are not experienced by CV endothelial cells. Indeed, our previously published findings show a high degree of pulsatility and cyclical change in diameter of the DA with blood flow pulsation during early development^2^. In vitro and in vivo studies have shown that KLF2 expression is differentially regulated by different types of flow regimes—typically showing increased expression by laminar flow and reduced expression associated turbulent, non-laminar flow^28,29,31,32,38,42^.

Although we show that developing primitive arteries show reduced KLF2 expression compared to primitive veins in both zebrafish and mice, the endothelium of mature arteries expresses high levels of KLF2^29,31,32,38^. How can the latter results be reconciled with our model (Fig. 7) in which “arterial” blood flow reduces KLF2 expression and the presence of KLF2 suppresses arterial-specific recruitment of vSMC? These results make sense if the expression of KLF2 is responsive to endothelial cell stretch/strain. Primitive arteries initially only consist of an endothelial layer without encircling smooth muscle layers, resulting in high levels of endothelial stretch and strain in response to pulsatile flow, as noted above. Gradual acquisition of vSMCs and assembly of vascular basement membrane layers creates resistance to the cyclical changes in vessel diameter with blood flow pulsation, reducing the “stretch” experienced by endothelial cells. Again, our previously published results show reduced vessel wall movements and pulsatile blood flow-induced changes in diameter as the DA acquires vSMC^2^. Endothelial recruitment of vSMC is required for formation of a vascular basement membrane, and this is also critical for reducing vessel distensibility, and thus endothelial cell stretch/strain^1–3,49^.

Altogether, these data point to dynamic, flow-regulated expression of KLF2 as a mechanism not only for promoting arterial-specific recruitment of vSMCs, but also for self-limiting and self-tuning the extent of vSMC acquisition to match the specific hemodynamic requirements of different regions of the arterial vascular tree. As developing arteries acquire vSMC and vSMC-promoted basement membrane, reduced vessel compliance/increased stiffness would be predicted to result in increased KLF2 expression, creating a “stop signal” to halt further vSMC recruitment and promote vSMC differentiation and quiescence^46,47,67^. In larger and/or more proximal arteries experiencing more dramatic cyclic flux in blood pressure, the “stop signal” might be expected to come later, after acquisition of a greater amount of vSMC and matrix and formation of a thicker and more robust vessel wall. Thus, this mechanism would ensure that the capacity of the vascular wall to resist stretch matches the flow regime vessels experience. Although this seems like a very attractive mechanism to allow arteries to “self-regulate” vSMC acquisition based on intrinsic flow dynamics, additional experimental studies will of course be needed to further substantiate that this is indeed an important determinant of the extent of vascular wall assembly.

A number of disease states have known links to altered blood flow sensing and/or are correlated with altered Klf2 expression profiles, including atherosclerosis and Cerebral Cavernous Malformations (CCMs), and it seems possible that the KLF2-CXCL12/CXCR4-PDGFB molecular pathway that we have identified for arterial vSMC acquisition during development may also play critical roles in vascular pathologies. As noted in our introduction, sites of low Klf2 expression are correlated with atherosclerotic lesion formation^23,28,36,38^. The experimental data from our study showing that low Klf2 expression correlates with high chemokine and high PDGFB expression suggests that suppressed Klf2 could be promoting atherogenesis at least in part by promoting chemokine/PDGFB-stimulated vSMC proliferation and reactivated motility^46,47,65–67,74–76^. Altered chemokine signaling has already been implicated in coronary artery disease, mobilization of progenitor cells in ischemia induced remodeling/repair, and atherosclerosis^59,64–68^. Upregulated chemokine signaling can lead to increased smooth muscle cell proliferation and chemotaxis, and it is known that vSMCs actively de-differentiate and invade atherosclerotic plaques^65,66^. Our results would suggest that the effects of chemokine signaling on the motility and proliferation of vSMCs in these pathological contexts may be indirect, through upregulation of PDGFB. Other disease models also offer some support for this idea.

CCM pathology, on the other hand, is associated with high Klf2/4 expression^77–79^. CCM is a vascular malformation disorder particularly localized to intersection points between capillaries and veins that leads to the formation of large dilated vessels^77–80^. These dilated vessels are prone to rupture, leading to trauma, tissue damage, and ischemia. Formation of CCM lesions has been linked to mutations in one of three different CCM proteins, that appear to have their deleterious effects at least in part via a Cdc42-MEKK3-MEK5-ERK5-KLF2/4 signaling cascade within the endothelium^36,38,78–80^. Vessels in CCM lesions have low numbers of associated vSMC/mural cells, consistent with the idea that high Klf2 levels serve as a “stop” signal for vSMC recruitment. It will be interesting to further determine whether vSMC recruitment and the KLF2-CXCL12/CXCR4-PDGFB pathway we have identified are involved in a causative way in lesion formation, or only secondarily affected as a result and/or part of developing CCM lesion pathology.

In closing, we describe a signaling pathway that promotes association of vSMCs with the arterial vasculature and restrains association with the venous vasculature during development. This pathway links blood flow, through the transcription factor Klf2, to chemokine signaling, PDGFB production, and ultimately arterial endothelium-directed motility of vSMCs. In addition to its important role in arterial/venous development, this signaling cascade may also be involved in vascular pathologies that alter vSMC acquisition or function.

## Methods

### Zebrafish methods and zebrafish transgenic lines

Zebrafish *(Danio rerio)* embryos were raised and maintained as described^81,82^. Zebrafish husbandry and research protocols were reviewed and approved by the NICHD Animal Care and Use Committee. Zebrafish transgenic lines Tg*(kdrl:mCherry-CAAX)*^*y171*^; Tg(*tagln:egfp*)^*p151*^ are previously published. New CRISPR/Cas9 mutant alleles generated for this manuscript include: (*cxcl12b*^*Δ24*^*)*^*y611*^; *(cxcr4a*^*Δ7*^*)*^*y612*^; *(pdgfbb*^*Δ3*^*(chromosome3))*^*y613*^; *(pdgfba*^*Δ6*^*(chromosome22))*^*y614*^; *(klf2a*^*Δ8*^*)*. ^*y616*^

### Reagents

Antibodies for immunostaining and western blot analysis include: Tubulin (Sigma, #T6199- 1:10,000 dilution); PECAM-1 (CD31) (BD Pharmingen; #553370- 1:300 dilution); alpha-sma-cy3 (Sigma; #c-6198; 1:500 dilution); Sm22 (GeneTex; #GTX101608; 1:300 dilution); PDGFB (SantaCruz; #sc-365805; 1:500 dilution); CXCR4 (Sigma, #SAB3500383; 1:1000); CXCL12 (R&D Systems, #AF-310-NA, 1:1000). WISH probes utilized include *klf2a*^20^, *pdgfba*^83^, *cxcr4a*^55,60^, and *cxcl12b* (Primers FW: GAGCTCTGGACACTCGCTGT; RV: TACTGCTGAAGC CATTTGGTC).

### Imaging and microscopy

Fluorescent images were collected utilizing either the Leica SP5 II or Nikon Yokogawa CSU-W1 spinning disk confocal microscope at 5 dpf at x20 magnification. Embryos were immobilized in buffered MS-222 and embedded in 0.8% low melting point agarose. All images were acquired, data quantified and analyzed blindly, and then embryos genotyped.

### In silico database analysis

We developed a “short list” of arterial-enriched candidates to study by (i) searching the zebrafish ZFIN expression database (https://zfin.org/action/expression/search) to identify genes expressed in the dorsal aorta but not expressed or expressed at lower levels in the cardinal vein during stages of vSMC recruitment (or vice versa), then pared down our candidates by (ii) identifying which of these genes were implicated in cell-cell signaling, and (iii) identifying which of these genes has been previously implicated in vascular signaling. The *cxcl12b* ligand emerged as a clear, strong candidate based on these criteria.

### Measurements of vSMC number and dorsal aorta width

#### Assessing vSMC Number

For each individual embryo, we acquired z-stacks on a spinning disk confocal microscope (Nikon Yokogawa CSU-W1) taking stacks through the area of the dorsal aorta and cardinal vein vasculature. Each z-stack is then analyzed plane by plane to determine cell boundaries and vSMC cell number. As such, the schematics in Figs. 2 and 4 are included to provide examples of what are counted as cells under different conditions. Importantly, all of our imaging and data analysis is carried out prior to genotyping the embryos, and data is sorted post hoc. This allows us to implement unbiased standardization of imaging and fully blinded analysis of our zebrafish mutant datasets.

#### Dorsal aorta width measurements

All embryos are imaged and analyzed from z-stack max projections at 5 dpf unless otherwise indicated. Dimension of the images is set to be consistent between all samples. Ten diameter measurements are then taken, at equidistant locations across the length of the vessel image. These measurements all are all represented independently within the final analysis. All measurements are taken prior to the authors knowing the genotype of the animal, and the data is sorted into groups post genotyping. As such, the measurements were collected in a completely blinded fashion. The final data in the graphs are standardized to sibling matched controls, and data is included from at least three independent clutches of embryos.

### Endothelial cell culture and 3-D assays

Human umbilical vein endothelial cells (HUVEC, Lonza) were cultured in bovine hypothalamus extract, 0.01% Heparin and 20% FBS in M199 base media (Gibco) on 1 mg/ml gelatin coated tissue culture flasks. HUVECs were used from passages 3–6.

Human coronary artery smooth muscle cells (CASMC, Lonza) were cultured in 10% FBS in Advanced DMEM base media (Gibco) on 1 mg/ml gelatin coated tissue culture flasks. PASMCs were used from passages 3–8.

Three-dimensional (3-D) collagen type I in vitro assays were done essentially as described^3,73^, utilizing 2.5 mg/ml collagen type I (BD Biosciences, Acid Extracted) gels including CXCL12 (R&D Systems, #350-NS/CF) or PDGFB (R&D Systems, #220-BB/CF) at 100 ng/ml. CASMCs were seeded on the collagen gel at 40,000 cells per well density. Culture media for the assays contained ascorbic acid, FGF (R&D Systems, #233-FB-025/CF), and IGF-II (R&D Systems, #292-G2-250). Assays were fixed in 2% paraformaldehyde (PFA) at 2 days and processed for future analysis.

#### siRNA transfection

Invitrogen SilencerSelect validated small-interfering RNA (siRNA), targeting CXCR4#1 and #2 (all quantification in the manuscript is shown from CXCR4 siRNA Target #1), CXCL12, and KLF2 were purchased and resuspended in H2O at a concentration of 40 μM. siPORTamine (LifeTechnologies/Ambion) was used as the transfection reagent. A double transfection protocol was used with a final concentration of 50 nM siRNA added per transfection^73^.

### CRISPR/Cas9 generation of zebrafish mutants

Mutations in the zebrafish *cxcl12b, cxcr4a, pdgfbb, pdgfba*, and *klf2a* genes were generated using the CRISPR/Cas9 system. The following guide RNAs were transcribed in vitro using the T7 mMessage Machine® Kit (Ambion), and injected at a dose of 150 pg/nl per embryo:

*cxcl12b*^*Δ24/Δ24*^: TAATACGACTCACTATAGGAGCCCAGAGACTGACGGTGTTTTAGAGCTAGAA

*cxcr4a*^*Δ7/Δ7*^: TAATACGACTCACTATAGGACATCGGAGCCAACTTTGGTTTTAGAGCTAGAAATAGCAAG

*pdgfbb*: TAATACGACTCACTATAGGCTGTGGTTGAGTTGGTGAGTTTTAGAGCTAGAA

*pdgfba*: TAATACGACTCACTATAGGACCCTCTTCCTCCATCTCGTTTTAGAGCTAGAA *klf2a*^*Δ8/Δ8*^: TAATACGACTCACTATAGGTCCGTAACTATCCATGCAGTTTTAGAGCTAGAAATAGCAAG

pT3TS-nCas9 (Addgene) was transcribed using MEGA Script T7 kit (Invitrogen/Ambion), and injected at a dose of 300 pg/nl per embryo. Embryos were injected at the single cell stage, screened for cutting efficiency and grown on system. F1 generations were analyzed for mutations, and pairs crossed for analysis in the F2 and beyond generations.

### Genotyping of zebrafish mutants

Mutants were genotyped using the following primers:

cxcl12b^Δ24^:

FW: TGTAAAACGACGGCCAGTGTATCACTTATATTCTCAAC

RV: GTGTCTTCACTCGCTCTTGGCATGGATAGC

cxcr4a^Δ7^:

FW: TGTAAAACGACGGCCAGTCAGCACATCGTCTTTGAAGATGATTTATC

RV: GTGTCTTGGCAGAGTGAGCACAAACAGAAGG

pdgfb ^Δ4^ (chromosome 3):

FW: TGTAAAACGACGGCCAGTGATTGTTTGATTAATAAGGAC

RV: GTGTCTTCTACAACATGTGACAAATTC

pdgfa ^Δ8 or Δ20^ (chromosome 22):

FW: TGTAAAACGACGGCCAGTAGGTGTTGTTTTGTTCAGGACC

RV: GTGTCTTTGGTATGGGATCAGCTTTACCT

klf2a^Δ8^:

FW: TGTAAAACGACGGCCAGTGACATTGACACCTACTGC

RV: GTGTCTTGAGTCATGCTGCCTGCTCC

Universal primer: FAM-M13: 5′-/56-FAM/TGTAAAACGACGGCCAGT -3′

### ABI 3130xl fragment analyzer protocol

PCR protocol with AmpliTaq Gold DNA Polymerase 1x (10µl) Rxn: 1 µl 10x PCR Gold Buffer; 0.5 µl MgCl2 25 mM; 1 µl 0.5 mM ABI Fwd primer; 1 µl 1 mM ABI Rev primer; 0.2 µl 10 mM FAM-M13 primer; 0.1 µl dNTP Master Mix; 0.1 µl TaqGold polymerase; 1 µl of 1:10 diluted crude gDNA; 5.1 µl H20.

#### TaqGold PCR Program

95 °C 10 minʹ 95 °C 30 s; 58 °C 30 s; 72 °C 30 s (1 min/kb); GoTo Step 2 × 34; 72 °C 10 min; 15 °C Hold; Run on ABI immediately or store at 4 °C in the dark for 24 h max.

#### ABI 3130xl plate set-up

HiDi Formamide/ROX master mix-0.2 µl ROX400HD; 9.8 µl HiDi Formamide; add 10 µl of master mix to each ABI plate sample well; add 2 µl of fluorescent PCR product; cap wells and denature at 95 °C for 5 min; uncap all wells and replace with ABI plate septa to run on the 3130xl. Follow manufacturer directions to utilize the ABI 3130xl.

### Mouse lines, breeding, and genotyping

#### Cxcr4 knockout mice

We initially generated *Cxcr4*^+/−^ heterozygous mice from the breeding of *Cxcr4-flox* mice (Jax# 008767) with *E2a-Cre* mice (Jax#003724). *Cxcr4*^−/−^ homozygous embryos were produced by mating *Cxcr4*^+/−^ heterozygous mice. Offspring were genotyped by genomic PCR using primers that specifically detect *Cxcr4* allele and *Cxcr4* null allele.

*Cxcr4* allele (347 bp):

FW: CAC TAC GCA TGA CTC GAA ATG

RV: GTG TGC GGT GGT ATC CAG C

*Cxcr4* null allele (190 bp):

FW: CAC TAC GCA TGA CTC GAA ATG

RV: CCT CGG AAT GAA GAG ATT ATG C

Klf2-GFP mouse was published previously^84^, and is a knockin allele where the GFP is fused to the N-terminus of KLF2. GFP signal was amplified using a GFP antibody (Goat anti-GFP, Abcam, CAT#ab6673 1:250) and the endothelium labeled with PECAM-1 antibody (Dianova, CAT# DIA-310 1:200).

### Immunostaining and western blot analysis

Tissue sections were immunostained following the same basic protocol: (1) 30 min room temperature (RT) incubation in Tris-Glycine; (2) 1 h RT incubation +/− permeabilization with 0.01% TritonX-100; (3) 2 h RT incubation in blocking solution (5% Sheep Serum, 1% Roche Blocking Buffer in PBST); (4) 1 h at RT—overnight 4 °C incubation with 1:1000 primary antibody unless otherwise noted; (5) wash with PBST; (6) 2–3 h RT incubation with 1:2000 secondary antibody in 5% Sheep Serum, 1% Roche Blocking Buffer in PBST; (7) wash with PBST and imaging analysis. Quantification of immunostaining intensity was performed by ImageJ analysis software. Images were acquired using a Leica SP5 II confocal microscope or Nikon Yokogawa CSU-W1 spinning disk confocal microscope. All images were acquired at the same intensity, step size and image resolution for analysis. The images were analyzed using ImageJ^85^. Data is reported as the percent average intensity per region of interest size from a minimum of three images from three independent experiments/animals ± s.e.m.

Zebrafish samples for western blot analysis were deyolked and directly lysed in 2x Laemmli Sample Buffer containing 5% b-ME and a PhosSTOP tablet (Roche), 10 µl per embryo unless otherwise indicated. HUVEC cells for Western blot analysis were lysed directly in 2x Laemmli Sample Buffer containing 5% b-ME and a PhosSTOP tablet (Roche), 500 µl per T-25 culture flask.

Secondary Digital-HRP-conjugated antibodies were purchased from Kindle Bioscience and used at 1:1000 in 5% milk. Primary antibodies are described in the “Reagents” section and used at 1:1000 unless otherwise noted. Images were acquired using the Kindle Bioscience KwikQuant Imager and 1-Shot Digital ECL. Quantification of relative band density was performed using ImageJ software. Data is reported as the percent average density from a minimum of 2–3 blots from at least two independent experiments ± s.e.m.

### qPCR and RNA extraction

Zebrafish embryos or HUVECs were collected at the indicated time points in TRIZOL and RNA purified using a double chloroform extraction protocol. cDNA was generated using BioRads I-Script cDNA synthesis kit from 500 ng of RNA. TaqMan qPCR protocols were utilized to generate relative expression data, and analysis run using the FAM channel of a 96-well BioRad CFX qPCR machine. Primer product numbers are as follow:

PDGFB (human): Hs00966522_m1

CXCL12 (human): Hs03676656_mH

KLF2 (human): Hs00360439_g1

CXCR4 (human): Hs00607978_s1

Ef1a (human): Hs00265885_g1

GAPDH (human): Hs02758991_g1

### Statistics and reproducibility

Statistical analysis of data was done using Microsoft Excel or “R.” Statistical significance was set at a minimum of *p* ≤ 0.05 and is indicated in individual figures. Student’s *t*-tests (Excel) were used when analyzing two groups within a single experiment, and was the standard method utilized unless otherwise noted. All statistics shown assess the treatment versus the control for each experiment. One-way ANOVA when controlling for type 1 errors (Excel) and two-way ANOVA with unequal sample size when controlling for sample interdependence (“R”). Normality of data was assessed via kurtosis and skewness measurements. Bar graphs were generated with Plotly.

### Study approval

Zebrafish husbandry and research protocols were reviewed and approved by the NICHD Animal Care and Use Committee at the National Institutes of Health. All animal studies were carried out according to NIH-approved protocols, in compliance with the *Guide for the Care and use of Laboratory Animals*.

### Reporting summary

Further information on research design is available in the Nature Research Reporting Summary linked to this article.

## Supplementary information

Supplemental Information

Description of Additional Supplementary Files

Supplementary Movie 1

Supplementary Movie 2

Supplementary Data 1

Reporting Summary

## Data Availability

All data reported in this manuscript are available from the corresponding author upon request. Data underlying all charts and graphs are available online in the associated “Source Data” file.
