# Supplementary material for: Chemokine mediated signalling within arteries promotes vascular smooth muscle cell recruitment
Source: Commun Biol. 2020 Dec 4;3:734. doi: 10.1038/s42003-020-01462-7 (PMC7719186; doi:10.1038/s42003-020-01462-7)
Supplement: Supplementary file 1 — Supplemental Information [file 42003_2020_1462_MOESM1_ESM.pdf]

Supplementary Information
Stratman, et. al.

Stratman, et al. Supplemental Figure 1

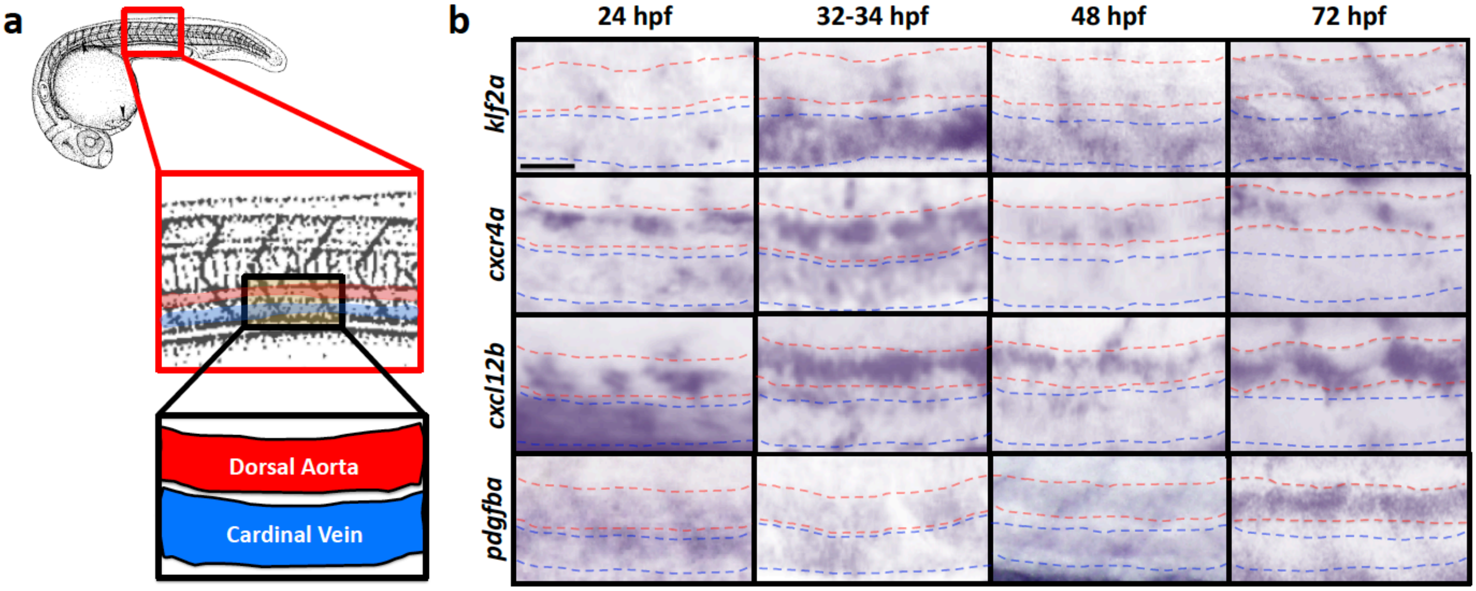

Supplementary Figure 1. In situ hybridization expression profiles of *klf2a*, *cxcr4a*, *cxcl12b*, and *pdgfba*. **A**, Schematic representation of the area imaged for *in situ* hybridization analysis, demonstrating the location of the dorsal aorta (DA) and cardinal vein (CV) within these images. **B,** Whole mount *in situ* hybridization (WISH) images of *klf2a, cxcr4a, cxcl12b,* and *pdgfba* transcript (purple) in 24 hpf, 32-34 hpf, 48 hpf, and 72 hpf zebrafish. Red dashed lines outline the dorsal aorta, blue dashed lines outline the cardinal vein. Time points are presented vertically, and genes of interest are presented horizontally. Scale bar = 75 um.

Stratman, et al. Supplemental Figure 2

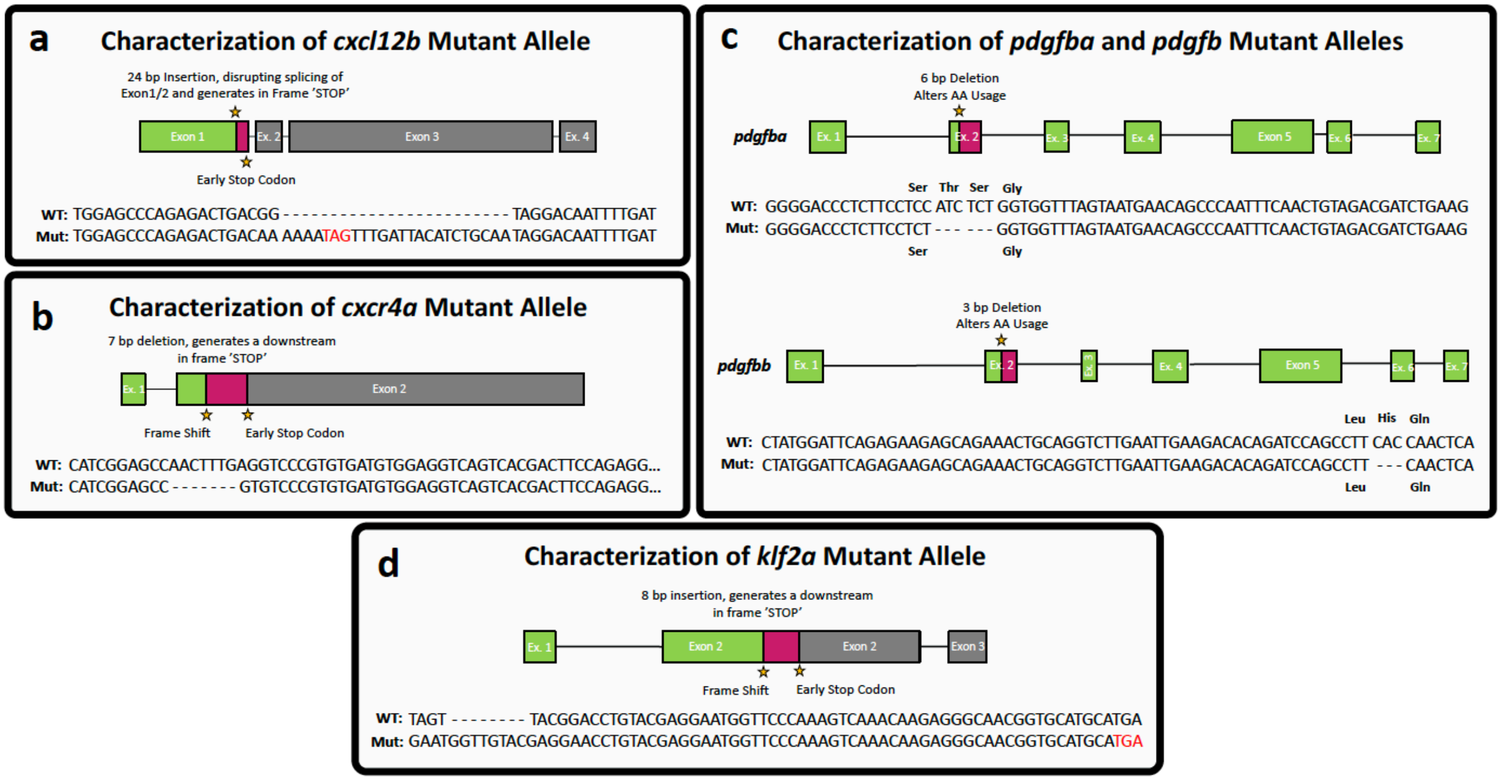

Supplementary Figure 2. CRISPR/Cas9 mutant alleles. Gene structure and sequence of mutants generated for this study using CRISPR/Cas9 mutagenesis. **A, *cxcl12b*$^{\Delta 24}$ mutant.** A 24 base pair insertion was introduced that destroys the splice donor site between exons 1 and 2 and inserts an in frame early stop codon. **B, *cxcr4*$^{\Delta 7}$ mutant.** A 7 base pair deletion in exon 2 leads to a frame shift at amino acid 75 and early termination after amino acid 198. **C, *pdgfba*$^{\Delta 6}$ mutant.** A 6 base pair deletion in exon 2 (chr22:29,325,360-29,325,754) leads to an in-frame deletion of two polar amino acids-a threonine and serine- leading to predicted alterations in protein folding. **D, *pdgfbb*$^{\Delta 3}$ mutant.** A 3 base pair deletion in exon 2 that leads to an in-frame deletion of a histidine leading to predicted alterations in protein folding. **E, *klf2a*$^{\Delta 8}$ mutant.** An 8 base pair insertion in exon 2 leads to a frame shift at amino acid 476 and early termination after amino acid 543.

Stratman, et al. Supplemental Figure 3

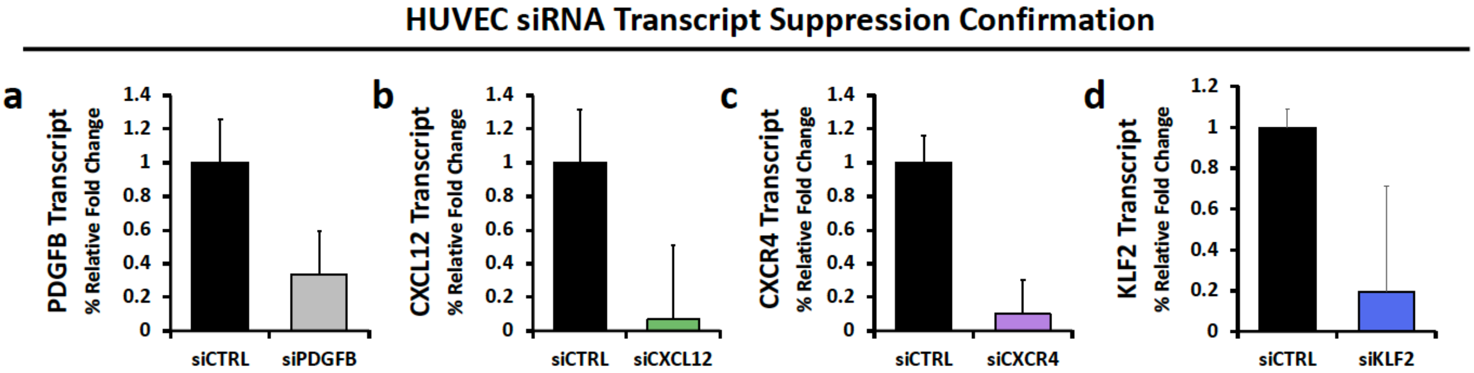

Supplementary Figure 3. siRNA knockdown in Human Umbilical Vein Endothelial Cells (HUVEC). We confirmed suppression of genes targeted by siRNAs in HUVECs for this study. Transcript levels were measured by performing qRT-PCR for each respective gene, comparing control siRNA treated to PDGFB (**A**), CXCL12 (**B**), CXCR4 (**C**), or KLF2 (**D**) siRNA treated cell samples. Data are representative of 3 experimental replicates. Values show the relative fold change compared to the control siRNA levels. Error bars ± s.d.

Stratman, et al. Supplemental Figure 4

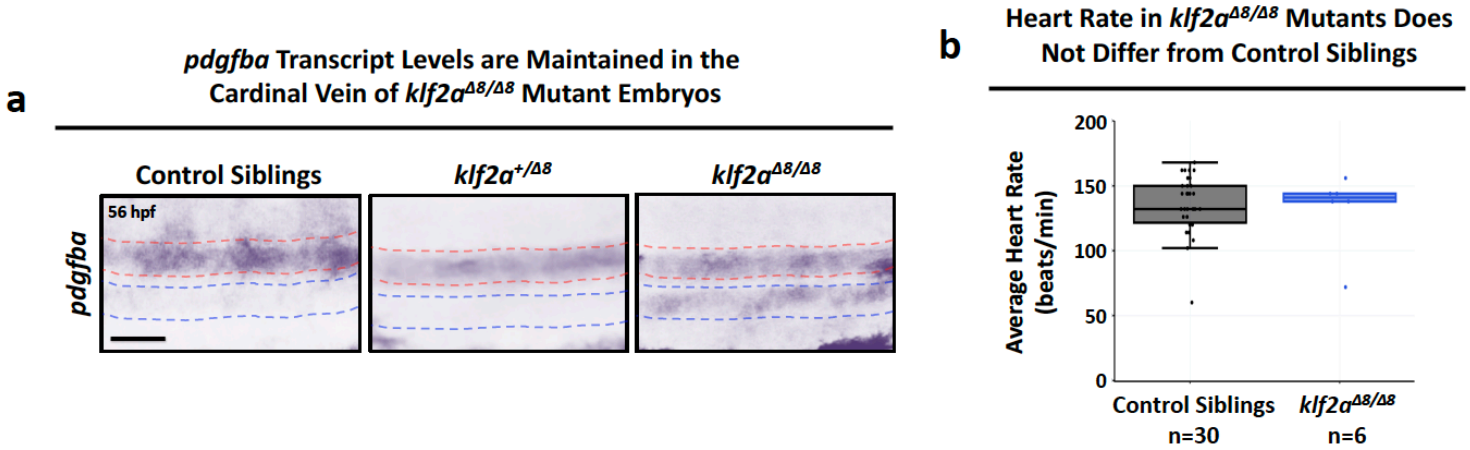

Supplementary Figure 4. *klf2a* genetic mutants maintain upregulated *pdgfba* transcript levels. A, Representative whole mount *in situ* hybridization (WISH) images of 56 hpf wild type, heterozygous, and homozygous *klf2a*$^{\Delta 8/\Delta 8}$ zebrafish mutants probed for *pdgfba*. Red dashed lines indicate the dorsal aorta; blue dashed lines indicate the cardinal vein. Scale bar = 75 um. **B**, Heart rate measurements in control siblings versus *klf2a*$^{\Delta 8/\Delta 8}$ mutant embryos. The graph demonstrates that the average beats per minute is unchanged between the two conditions. Box plots are graphed showing the median versus the first and third quartiles of the data (the middle, top, and bottom lines of the box respectively). The whiskers demonstrate the spread of data within 1.5x above and below the interquartile range. All data points are shown as individual dots, with outliers shown above or below the whiskers.

Stratman, et al. Supplemental Figure 5

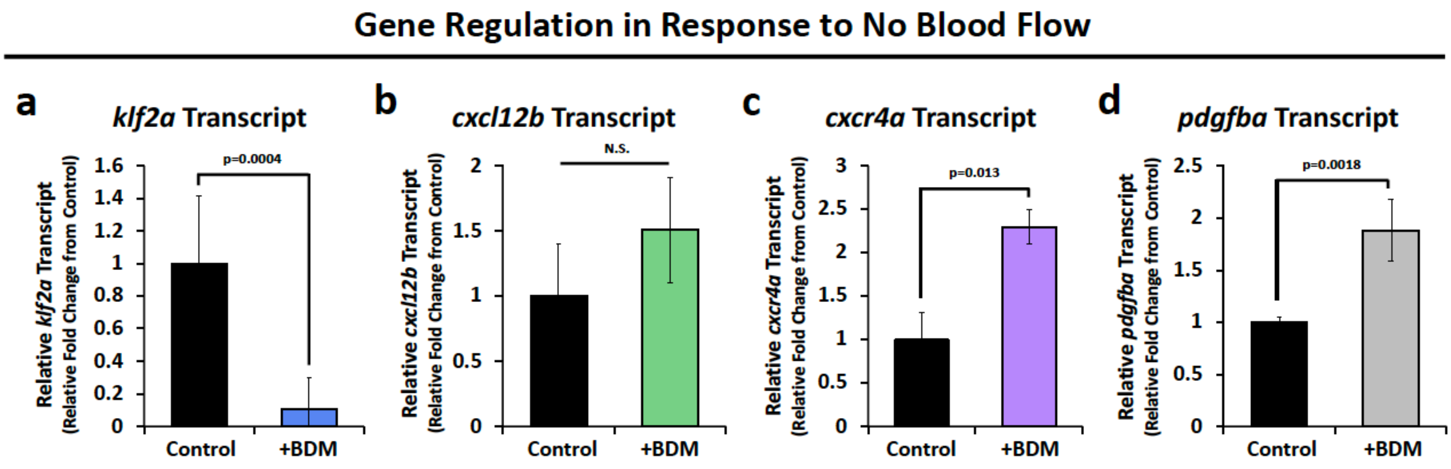

Supplementary Figure 5. qPCR analysis of pro-vSMC arterial recruitment cues following a transient stop in blood flow. Zebrafish were allowed to develop normally for 36 hpf, then divided into two groups. Group 1 were vehicle control treated embryos, Group 2 were treated with the chemical BDM (2,3-butanedione monoxime) for 12 hours to slow/stop the beating of the embryonic heart. Total RNA was extracted from the embryos at 48 hpf and analyzed by qPCR for gene transcript levels. All samples are expressed as a fold change from the control. (**A**) *klf2a,* (**B**) *cxcl12b,* (**C**) *cxcr4a,* and (**D**) *pdgfba.* Error bars ± s.d.

Stratman, et al. Supplemental Table 1

	Wild Type	Heterozygote	Homozygote
**Expected Ratio**	**25%**	**50%**	**25%**
cxcl12b	19.12	48.53	32.35
cxcr4a	22.86	47.14	30.00
klf2a	27.14	51.43	21.43
pdgfba	20.99	51.85	27.16
**Expected Ratio**	**0%**	**50%**	**50%**
pdgfbb	0.00	43.68	56.32

Supplementary Table 1. Genotypic ratios of zebrafish mutant lines. All zebrafish mutant lines were analyzed from in-crosses of heterozygous mutant carriers, except for the *pdgfbb* mutant line which was analyzed as an in-cross of a homozygous mutant to a heterozygous mutant. The expected mendelian ratios are shown for each line, as well as the recovered ratios. All embryos presented in the manuscript were phenotyped then genotyped, and are represented within the table.

Full Uncropped Western Blots from Main Figures

Figure 5b:

Samples (L to R) : control, 2hr, 4hr, 8hr, 22hr

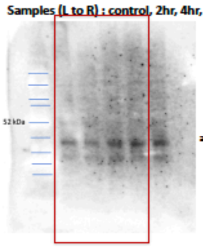

anti-pdgfbb (top band, 31-35kDa)

Samples (L to R): control, 2hr, 4hr, 8hr, 22hr

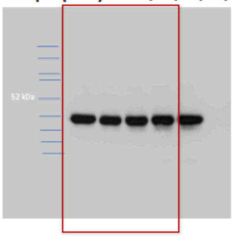

anti-GAPDH (37 kD)

Samples in red box presented in manuscript:
(L to R) control, 2hr, 4hr, 8hr

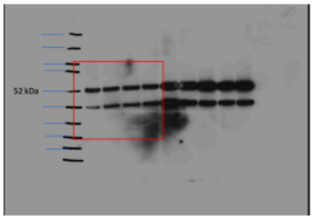

a-tubulin (top band, 50 kDa)

PDGFbb (lower band, 31-35kDa)

Figure 5d:

Samples: Control, Cxcr4 #1, Cxcr4 #2, Cxcl12

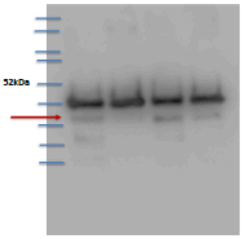

anti-pdgfbb (arrow, 31-35kDa)

Samples: Control, Cxcr4 #1, Cxcr4 #2, Cxcl12

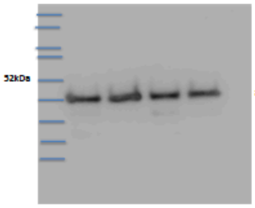

anti-GAPDH (37 kD)

Samples in red box presented in manuscript: Control, Cxcr4 #1, Cxcr4 #2, Cxcl12

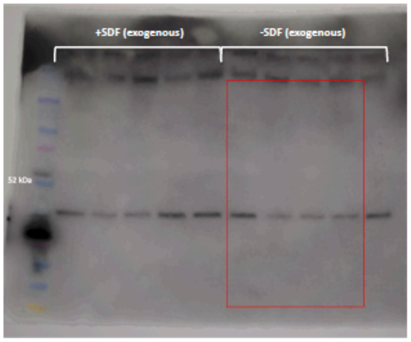

anti-Pdgfbb (31-35kDa)

Samples in red box presented in manuscript: Control, Cxcr4 #1, Cxcr4 #2, Cxcl12

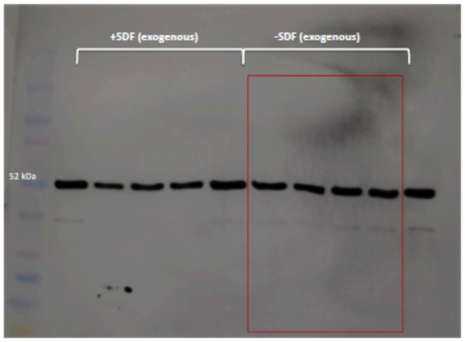

anti-Tubulin (50 kDa)

Figure 5i:

Samples: Control #1, Cxcl12 RNA #1, Control #2, Cxcl12 RNA #2

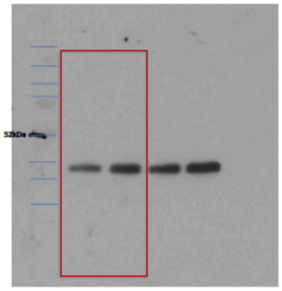

Lanes 1/2 presented in manuscript

Lanes 3/4 are a biological replicate

anti-Pdgfbb (31-35kDa)

Samples: Control #1, Cxcl12 RNA #1, Control #2, Cxcl12 RNA #2

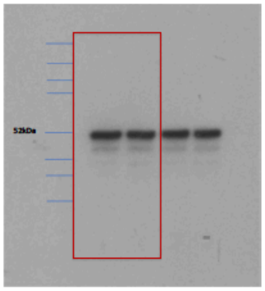

Lanes 1/2 presented in manuscript

Lanes 3/4 are a biological replicate

anti-Tubulin (50 kDa)

Figure 6f:

Samples: Klf2 siRNA, Control, CXCR4 siRNA

Only the first two columns are presented in the manuscript, cropped and flipped in the horizontal orientation

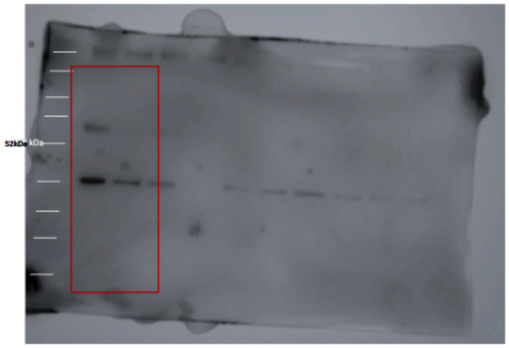

Top Band: CXCR4 (~60 kDa)

Lower Band: PDGFBB (31-35 kDa)

Samples: Klf2 siRNA, Control, CXCR4 siRNA

Only the first two columns are presented in the manuscript, cropped and flipped in the horizontal orientation

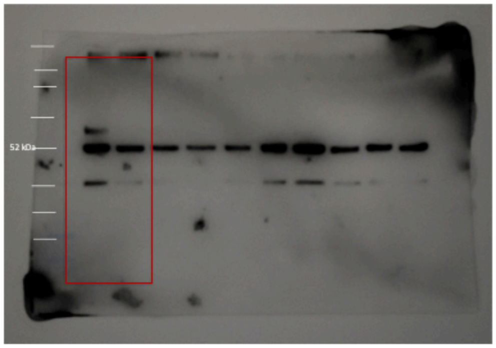

Tubulin (50 kDa)
