# Supplementary material for: Chemokine mediated signalling within arteries promotes vascular smooth muscle cell recruitment
Source: Commun Biol. 2020 Dec 4;3:734. doi: 10.1038/s42003-020-01462-7 (PMC7719186; doi:10.1038/s42003-020-01462-7)
Supplement: Supplementary file 2 — Description of Additional Supplementary Files [file 42003_2020_1462_MOESM2_ESM.pdf]

Description of Additional Supplementary Files

File Name: Supplementary Movie 1
Description: Live imaging of blood flow in klf2aΔ8/Δ8 wild type siblings. Images were acquired at 20 frames/second across a 15 second time course. Blood flow through the axial vasculature is readily observed.

File Name: Supplementary Movie 2
Description: Live imaging of blood flow in klf2aΔ8/Δ8 mutant embryos. Images were acquired at 20 frames/second across a 15 second time course. Blood flow through the axial vasculature is readily observed.

File Name: Supplementary Data 1
Description: Source data
