# Supplementary material for: Chemokine mediated signalling within arteries promotes vascular smooth muscle cell recruitment
Source: Commun Biol. 2020 Dec 4;3:734. doi: 10.1038/s42003-020-01462-7 (PMC7719186; doi:10.1038/s42003-020-01462-7)
Supplement: Supplementary file 6 — Reporting Summary [file 42003_2020_1462_MOESM6_ESM.pdf]

nature research

Corresponding author(s): Brant Weinstein

Last updated by author(s): Oct 19, 2020

Reporting Summary

Nature Research wishes to improve the reproducibility of the work that we publish. This form provides structure for consistency and transparency in reporting. For further information on Nature Research policies, see our Editorial Policies and the Editorial Policy Checklist.

Statistics

For all statistical analyses, confirm that the following items are present in the figure legend, table legend, main text, or Methods section.

n/a	Confirmed	
☐	☒	The exact sample size (*n*) for each experimental group/condition, given as a discrete number and unit of measurement
☐	☒	A statement on whether measurements were taken from distinct samples or whether the same sample was measured repeatedly
☐	☒	The statistical test(s) used AND whether they are one- or two-sided<br>*Only common tests should be described solely by name; describe more complex techniques in the Methods section.*
☒	☐	A description of all covariates tested
☐	☒	A description of any assumptions or corrections, such as tests of normality and adjustment for multiple comparisons
☐	☒	A full description of the statistical parameters including central tendency (e.g. means) or other basic estimates (e.g. regression coefficient) AND variation (e.g. standard deviation) or associated estimates of uncertainty (e.g. confidence intervals)
☐	☒	For null hypothesis testing, the test statistic (e.g. *F*, *t*, *r*) with confidence intervals, effect sizes, degrees of freedom and *P* value noted<br>*Give P values as exact values whenever suitable.*
☒	☐	For Bayesian analysis, information on the choice of priors and Markov chain Monte Carlo settings
☒	☐	For hierarchical and complex designs, identification of the appropriate level for tests and full reporting of outcomes
☒	☐	Estimates of effect sizes (e.g. Cohen's *d*, Pearson's *r*), indicating how they were calculated

Our web collection on statistics for biologists contains articles on many of the points above.

Software and code

Policy information about availability of computer code

Data collection	Microsoft Excel, ImageJ, Microsoft Powerpoint
Data analysis	Microsoft Excel, ImageJ, Microsoft Powerpoint, Plotly

For manuscripts utilizing custom algorithms or software that are central to the research but not yet described in published literature, software must be made available to editors and reviewers. We strongly encourage code deposition in a community repository (e.g. GitHub). See the Nature Research guidelines for submitting code & software for further information.

Data

Policy information about availability of data

All manuscripts must include a data availability statement. This statement should provide the following information, where applicable:
- Accession codes, unique identifiers, or web links for publicly available datasets
- A list of figures that have associated raw data
- A description of any restrictions on data availability

No accession codes, unique identifiers, or web links for publicly available datasets. There are no restrictions on data availability. All figures have associated raw data.

Field-specific reporting

Life sciences study design

All studies must disclose on these points even when the disclosure is negative.

Sample size	Sample sizes were chosen based on standards in the field and previously published literature.
Data exclusions	Abnormally developed fish were excluded from studies.
Replication	All experiments have a minimum of three biological replicates to assure the consistency of the results.
Randomization	Randomization is not relevant to this study. We are observing developmental phenotypes associated with naturally randomized genotypes distributed within genetic crosses.
Blinding	The majority of this work is done on zebrafish embryos, which are genotyped after phenotypes are documented and scored, allowing for full blinding of both analysis and interpretation of the data. In the instances where this is not possible, two independent lab members analyzed data sets for congruency in findings.

Reporting for specific materials, systems and methods

We require information from authors about some types of materials, experimental systems and methods used in many studies. Here, indicate whether each material, system or method listed is relevant to your study. If you are not sure if a list item applies to your research, read the appropriate section before selecting a response.

Materials & experimental systems

n/a	Involved in the study	
☐	☒	Antibodies
☐	☒	Eukaryotic cell lines
☒	☐	Palaeontology and archaeology
☐	☒	Animals and other organisms
☒	☐	Human research participants
☒	☐	Clinical data
☒	☐	Dual use research of concern

Methods

n/a	Involved in the study	
☒	☐	ChIP-seq
☒	☐	Flow cytometry
☒	☐	MRI-based neuroimaging

Antibodies

Antibodies used	Antibodies for immunostaining and western blot analysis include: Tubulin (Sigma, #T6199- 1:10,000 dilution); PECAM-1 (CD31) (BD Pharmingen; #553370- 1:300 dilution); alpha-sma-cy3 (Sigma; #c-6198; 1:500 dilution); Sm22 (GeneTex; #GTX101608; 1:300 dilution); PDGFB (SantaCruz; # sc-365805; 1:500 dilution); CXCR4 (Sigma, #SAB3500383); CXCL12 (R&D Systems, #AF-310-NA).
Validation	All antibodies were validated by the manufactures.

Eukaryotic cell lines

Policy information about cell lines

Cell line source(s)	Human umbilical vein endothelial cells (HUVEC, ATCC), Human coronary artery smooth muscle cells (CASMC, Lonza)
Authentication	The cell lines were not authenticated beyond the manufactures or lab's declaration.
Mycoplasma contamination	Cell lines were not tested for mycoplasma contamination
Commonly misidentified lines (See ICLAC register)	none

Animals and other organisms

Policy information about studies involving animals; ARRIVE guidelines recommended for reporting animal research

Laboratory animals	mus musculus, B6, males and females, less than 1.5 years of age; danio rerio, EK, males and females, up to embryonic to 6dpf
Wild animals	n/a
Field-collected samples	n/a
Ethics oversight	Zebrafish husbandry and research protocols were reviewed and approved by the NICHD Animal Care and Use Committee at the

National Institutes of Health. All animal studies were carried out according to NIH-approved protocols, in compliance with the Guide for the Care and use of Laboratory Animals.

Note that full information on the approval of the study protocol must also be provided in the manuscript.
